# Game Design in Mental Health Care: Case Study–Based Framework for Integrating Game Design Into Therapeutic Content

**DOI:** 10.2196/27953

**Published:** 2021-12-01

**Authors:** Panote Siriaraya, Valentijn Visch, Marilisa Boffo, Renske Spijkerman, Reinout Wiers, Kees Korrelboom, Vincent Hendriks, Elske Salemink, Marierose van Dooren, Michael Bas, Richard Goossens

**Affiliations:** 1 Faculty of Industrial Design Engineering Delft University of Technology Delft Netherlands; 2 Department of Psychology, Education and Child Studies Erasmus University Rotterdam Rotterdam Netherlands; 3 Parnassia Addiction Research Centre Parnassia Psychiatric Institute The Hague Netherlands; 4 Addiction, Development and Psychopathology Lab Department of Psychology University of Amsterdam Amsterdam Netherlands; 5 Centre for Urban Mental Health University of Amsterdam Amsterdam Netherlands; 6 Tilburg School of Social and Behavioural Sciences Tilburg University Tilburg Netherlands; 7 Social and Behavioral Sciences Utrecht University Utrecht Netherlands; 8 &RANJ Serious Games Rotterdam Netherlands

**Keywords:** design models, gamification, case studies, mental health, eHealth

## Abstract

While there has been increasing interest in the use of gamification in mental health care, there is a lack of design knowledge on how elements from games could be integrated into existing therapeutic treatment activities in a manner that is balanced and effective. To help address this issue, we propose a design process framework to support the development of mental health gamification. Based on the concept of experienced game versus therapy worlds, we highlight 4 different therapeutic components that could be gamified to increase user engagement. By means of a Dual-Loop model, designers can balance the therapeutic and game design components and design the core elements of a mental health care gamification. To support the proposed framework, 4 cases of game design in mental health care (eg, therapeutic protocols for addiction, anxiety, and low self-esteem) are presented.

## Introduction

There has been growing interest in the use of gamification in the field of health care. The concept of gamification proposes that motivational elements drawn from the field of game design could be applied to a positive effect in a nongame context such as health care [1,2]. For example, this approach has been used to help users manage symptoms related to diabetes [3] and allow patients with cancer to better understand their condition [4]. The field of mental health care in particular has seen increasing interest in the use of gamification, especially to enhance the effectiveness of psychological therapies, often by improving adherence and engagement in the therapeutic activities (see [5,6]). It is often essential for clients in treatment to adhere to their given therapeutic assignments [7], but in practice this is quite difficult [8]. Therefore, various gamified therapeutic activities have been developed and used for the treatment of conditions such as attention deficit hyperactivity disorder [9].

Incorporating game elements into existing mental health interventions can be difficult as designers must constantly take into account the opportunities and restrictions in both the domain of game design and mental health treatment [10]. For example, when modifying an underlying treatment activity based on a specific game design strategy, designers must determine in what way this can be done to provide enough *game value* (ie, to motivate or persuade clients to adopt specific healthy behaviors or change maladaptive behaviors), while justifying the potential impact on the *therapeutic value* (ie, the capacity to reduce psychological symptoms)*.* Adding more game elements to improve engagement results in a more enjoyable game experience; however, the underlying therapeutic aspect might be negatively influenced [10]. Arbitrarily adding game elements without careful consideration of the target therapeutic audience risks creating a gamification which alienates players [11,12], whereas introducing game mechanics without considering their influence on the relationship with the underlying therapeutic activity risks corroding the effectiveness of the therapeutic mechanisms themselves [13]. As such, when creating a mental health care gamification, it is extremely important to consider the balance and relationship between the game and therapy elements in gamification. Often, the very nature of such issues means that there is no clear-cut guideline for the integration of game design and therapy elements in gamified health interventions [14].

This paper aims to address this issue by providing a design framework to enhance both the conceptual and practical knowledge of gamification in mental health care. It is based on our reflections and practical experiences of designing and evaluating 4 different gamifications. These gamifications cover 2 intervention protocols commonly used in mental health treatment (ie, cognitive training and cognitive behavioral therapy) and 2 categories of disorders (ie, externalizing and internalizing disorders). More specifically, in this paper:

- We propose the concept of a “game world” versus “therapy world” experience and identified 4 components of a “therapy world” that could be gamified by design into a “game world” experience with increased user engagement. We then highlight 3 different strategies for how integrated game therapy worlds could be created using the 4 components.
- We created a design process framework to help designers analyze the procedures used in a therapeutic intervention and design the core elements of a mental health care gamification based on the concept of a core-game loop.
- To support our framework, we provide 4 case studies of gamifications of mental health care therapies and analyzed their design process in detail through our framework.

### Previous Research on the Design of Digital Games and Gamification in Mental Health Care

#### Overview

Previously, digital games that have been developed for mental health care often embodied similar characteristics as those found in fully functional entertainment games, although with a health-related purpose at its core [14]. Such games usually contain a carefully designed fully functioning “game space” with their own gameplay mechanics and interactive aesthetics (eg, [15-17]). In some cases the therapeutic tasks and activities themselves are embedded in a game-level structure and players would need to achieve various health-related objectives in order to progress through the game [15,16]. For example, in the SPARX game, users go through different levels that challenge them to acquire core skills that would help them better cope with depression [16]. In other cases, the game space itself is designed to provide awareness (often by encouraging players to reflect through gameplay) and persuades players to adopt and maintain a behavior that improves well-being [4,17]. An example of this could be seen in the Playsafety game where players are shown various scenarios that depict dangerous situations related to drug use and are shown the consequences of making the wrong decision in those scenarios [17]. Overall, the design process of these games generally resembles those that are used to develop serious games in other application domains, such as in education. To design such games, the designer first defines “serious objective or outcome” and then designs an engaging game space (including story flow, rules and interaction–feedback system, etc.) around the desired outcome (ie, the therapeutic goal) [18]. In these games, the original “serious” nongame content generally remains independent from the game space. As such, users might experience a separation between the game content and the nongame content when playing the game and could result in players becoming immersed in the game space while not being engaged with the serious content.

The more recent interests in gamification have led some designers to further examine ways in which game methods and design approaches can be integrated as part of a therapeutic process. Instead of creating a full game space to help motivate clients in mental health treatment to realize a therapeutic objective (which tends to be costly and time consuming), recent approaches in gamification suggest that perhaps discrete game elements could instead be applied to enhance existing tasks within the treatment process (see [19]). It is believed that this would help transform the experience of doing an otherwise mundane task into one which is more engaging for users [20]. A particular appeal of this approach is that it allows for various commonly used game mechanics (such as point systems and leaderboards) and game experience designs (challenge, competition, etc.) to be viewed as “templates,” which could be carefully tested and then used as a blueprint to provide gamelike experiences to enhance different therapeutic tasks (see [19]). In the health care field in particular, which values an evidence-based approach when designing interventions, this approach can be particularly appealing as the effect of different game design decisions can be empirically tested and more clearly understood (serious games employing a full-blown game space have traditionally been difficult to validate and examine [21]). Examples of such game mechanics and elements used that have been transposed to a therapeutic task include in-game reward systems (badges, tangible rewards, and social feedback), a narrative driven quiz system [22], and a gamelike audio and visual feedback system combined with a point-based reward system [13,23].

However, many researchers caution against the difficulties and pitfalls of this kind of “integrative” gamification approach. Some researchers argue against the practice of employing discrete game elements without carefully designing for the emerging player experience [18]. These researchers propose that gamification should be viewed more as a way to create emerging experience of playfulness instead of relying on template game mechanics [24]. More specifically, these researchers argue that focusing only on the mechanics themselves (eg, only arbitrarily adding in elements such as points, badges, and levels) risks striping the games from their most engaging aspects, as the “engagingness” of a game comes not only from a single mechanic, but also rather from a well-designed and thought-out combination of the game mechanics with other elements of the game (such as the rules and narration) and with the serious content itself to create a ludic system that allows users to experience meaningful play and have fun [24,25]. Such researchers posit that to design such a system, it is important to carefully consider the underlying context and understand the goals of users within the serious activity when integrating the game mechanics (possibly by employing a user-centered design approach) [11,26]. Merely adding game elements to a therapeutic activity without careful consideration of the interests of the targeted audience and the nature of the target activity risks the gamification becoming less relevant to the user interests and thus becoming ineffective in motivating users [11]. Overall, in the domain of mental health care in particular, there have been few proposed design models that show the process of how elements from game design could be integrated effectively with the content of a therapeutic activity.

#### Integrating Game Elements Into Therapeutic Activity Through a Game Therapy Worlds Concept

A key essence in the process of mental health care gamification is the question of how various elements from game design could be incorporated into an existing therapeutic activity to create a more engaging and (at least) equally effective treatment process. Key issues such as which game elements can be added without conflicting with the underlying therapeutic processes, which parts of the treatment can be mixed with game elements, and which parts of the therapeutic content should remain unchanged for the gamified therapy to be effective would need to be addressed. The difficulty of such a process is compounded further by the fact that the therapeutic activities themselves are often black boxes [21]. It is often unknown whether changing the procedure, rules, or interactions of a certain therapeutic activity (to make it more engaging) would also jeopardize the therapeutic effect.

To provide a better understanding of how game elements could be combined with existing therapeutic activities, we draw upon the notion of “worlds,” which represent the changing experiences of a person as we examine gamification design in past studies. This notion suggests that when playing an immersive game, players gradually leave their world of daily life and move toward a fun and engaging game world [27]. Their experience changes and they feel “transported” [28] into a world of play. Huizinga [27] described this process as “creating a temporary “game” world within the ordinary world” for which he proposed the metaphor of a “magic circle” having its own time and space boundaries and absorbing the player “intensely and utterly.” It is exactly this absorbing and engaging quality of game experiences that can be used to enrich mental health care therapies. As such, in our “game therapy worlds” concept, we view games developed for mental health care as similarly consisting of 2 worlds: the game world, which denotes how users experience, interact, and are affected by the designed game experience; and the therapy world, which denotes the experiences, interactions, and effect of the designed therapeutic process within the mental health care game.

#### Therapy Games Formed From Separated Game and Therapy Worlds

**Figure 1:**
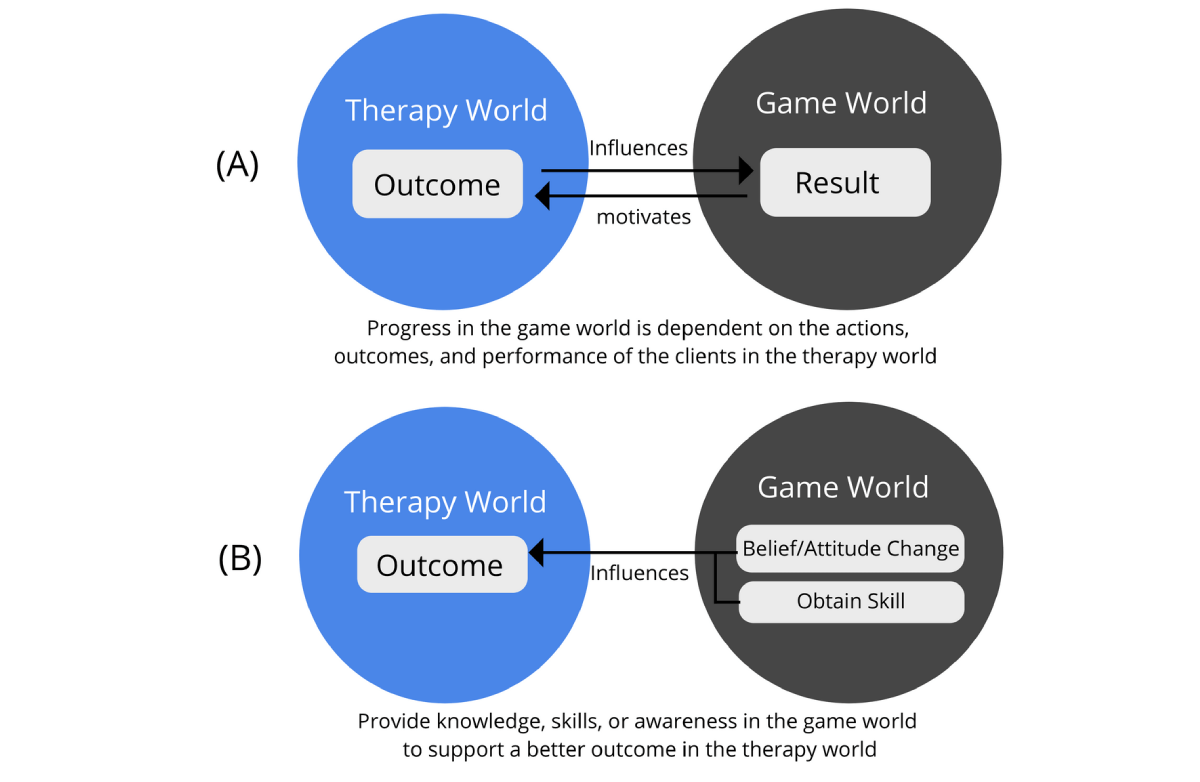
Strategies for incorporating a separate game world experience into the therapy world.

Two strategies have been commonly used in previous studies when designing games for mental health care. The first involves creating a discrete game world (with its own rules, narration, and interaction mechanics), where the performance and actions of users in a therapeutic activity are used to drive their progress in the game world (Figure 1A). An example of such strategy is the Changamoto game, which was developed to encourage youth to fill in a diary for their triggers in cannabis addiction. In the Changamoto game, a separate turn-based strategy game world was created in which players would need to command their own robot team to defeat an opposing team. Progression in the game is dependent on players successfully filling in entries in their diary [29].

The second strategy is to create a discrete game world to provide users with the knowledge and awareness to improve the outcome of an existing treatment (Figure 1B). An example of this is the Re-mission game, which was designed to provide children with knowledge related to their treatment and in doing so improve their adherence to the treatment [4]. The game itself was designed as a third-person action game with the interactions inside the game (ie, destroying cancer cells with weapons such as chemotherapy) designed to make players more aware and understand the importance of complying with their treatment regime.

In both strategies, the game world itself is usually not built upon an existing therapeutic activity but as a separate game space with its own rules and mechanics. The advantage of such a strategy is that there is only a limited relationship between the therapy world and the game world, and therefore there is much less potential for the game world to impact the targeted therapeutic activity. In addition, the engagement potential of the newly created game world is not hindered by the therapy rules, thus allowing for more flexibility when designing the game. These discrete game worlds often function as fully fledged games, containing their own mechanics, rules, and interactions, which are separate from the therapy. Players experience first the game world and then the therapy world. However, the disadvantage of this strategy is that the effectiveness of such games often depends on the entertainment quality of the game world itself and it could be quite costly to develop fully fledged games that are entertaining enough to be effective [14].

#### Therapeutic Games Formed From Integrated Game and Therapy Worlds

##### The Elemental Tetrad

Instead of creating a discrete game world to encourage users to achieve a specific therapeutic outcome, different aspects within the therapeutic activity itself could be transformed to become more engaging by using specific elements and approaches drawn from the field of game design [26]. Although there have been different suggestions of what components exist in a game (such as [30,31]), the concept of the elemental tetrad proposed by Schell [32] provides a useful distinction of the broad elements found in a game system. This tetrad proposes 4 interconnected game elements: the mechanics (the procedures, rules, and possible interaction space in the game), the aesthetics (the visual and audio qualities of the game), the technology (the medium in which a game has been implemented), and storyline (the prescripted events that emerge from the user interaction) [32]. While there are serious games in mental health care that have used technology (eg, virtual reality to create a more engaging experience [33]), aesthetics (eg, visualizing the consequence of how different bodily organs are affected by drugs [34]), or the storyline (eg, a game where users play the role of an investigator to learn about the consequences of drug overdoses [35]) as the core element, most gamifications focus on the use of the game mechanics to enhance the underlining experience (such as adding a point base system to create a sense of challenge) with the technology, aesthetics, and storyline being designed around the game mechanic elements [36]. Therefore, we focus on this aspect in our design framework as we analyze the components of an integrated therapy and game world.

##### The Components of an Integrated Therapy and Game Worlds

To allow us to better understand how a specific game element can be combined with the different aspects of the therapeutic procedure, we look at the components of a therapeutic activity from a game systems perspective from the viewpoint of the game mechanics element proposed by Schell [32]. When viewed from this perspective, there are many aspects of a therapeutic task that are similar in nature to the mechanics of a game system. For example, in a therapeutic activity, clients are generally presented with a list of tasks with different content (referred to as *content samples*) that they would need to resolve to achieve success in the therapy (eg, in the form of homework and out-of-session activities, which are common tasks in psychotherapy and cognitive behavioral treatment procedures [37,38]). These tasks are usually presented in a *structured* format (eg, Clark’s model [39] proposes that the situations that clients would confront as part of their social phobia treatment be presented in a hierarchical manner based on difficulty). In each task, users are presented with a meaningful choice where they can decide to take action to complete the task, after which feedback is provided by the therapist to help clients evaluate their performance (referred to as the *performance space*). An example could be seen from an Interpretation Bias Modification training procedure for social anxiety, where clients would choose whether a word representing a threat or benign interpretation is related to an ambiguous situation and would receive positive feedback when they relate benign interpretations toward ambiguous sentences [40]. Whether or not the user is successful in his/her task is determined by the *rules* of the therapeutic activity. For example, the retraining exercises which are used to change maladaptive cognitive biases based on the Dual-Process model of addiction often have specific rules that would need to be followed to be effective (such as to make avoidance actions in response to alcoholic stimulus within a set period) [41]. The cumulative completion of each therapeutic task is expected to result in a better health-related outcome. Overall, Table 1 provides an overview of the similarities between the components in the game world and the therapy procedures and provides examples of several treatment approaches and therapeutic activities where these components could be identified.

**Table 1:** Components of a therapeutic activity when viewed from a game systems perspective.

Component to be balanced	In a game world	In a therapeutic world
Performance space	The actions or meaningful choices available to players to act upon. Feedback is given to users as indicators to convey the results of their actions in the game.	The actions and choices are presented to the clients in a therapeutic activity, which can be acted upon to achieve success in the therapy. Feedback is conveyed to clients to report the results of their actions and is generally aimed at providing guidance in future choices. Example: *In contingency management programs, clients are presented with a task to abstain from drug use within a set period. They are presented with 2 choices of potential actions that could be taken: either choose to use drugs or not to use drugs within the set period. If clients choose not to use drugs, they would be presented with monetary rewards as a form of feedback to reinforce their positive behavior* [42].
Structure	Referred to as the “steps of play,” the structure component highlights the progression of players in the game [31].	A structured organization of tasks presented to guide clients to progress through the therapy. Example: *As part of weekly homework assignments in the cue exposure therapy to treat social phobia, clients are exposed to different social situations that they normally avoid. These situations are presented in a hierarchal structure based on their difficulty to help clients build their confidence, with easier tasks being presented before more difficult ones* [39].
Rules	The principles that govern the consequences of user actions and results within the game.	The rules or principles set by the therapy procedure that relate to the success or failure in achieving the health-related outcome. Example: *In the selective response inhibition training module used as part of the cognitive bias modification training to treat alcohol addiction, clients are presented with images representing alcoholic and nonalcoholic beverages. Clients would need to respond when shown images of nonalcoholic beverages in a timely manner by pressing a key and withhold their response when shown images of alcoholic beverages. Responding to alcoholic beverages or not responding in time results in a failure* [43].
Content Sample	The different events given to players through the game that they must overcome to achieve success. Referred to as a pulse [31].	The different content samples drawn from a therapeutic activity framework that are presented to the clients, who need to resolve them to achieve success in the therapeutic activity. Example: *In the interpretation bias modification training used to treat anxiety, clients are presented with ambiguous scenarios representing anxiety-related situations. Each scenario ends with a word fragment which clients need to solve toward a positive solution. Clients need to go through a series of multiple different scenarios (ie, content sample) representing different anxiety-related situations and complete the word fragment in each one, reinforcing a beneficial interpretation of ambiguous information* [44].

##### Strategies for Creating an Integrated Therapy and Game World

**Figure 2:**
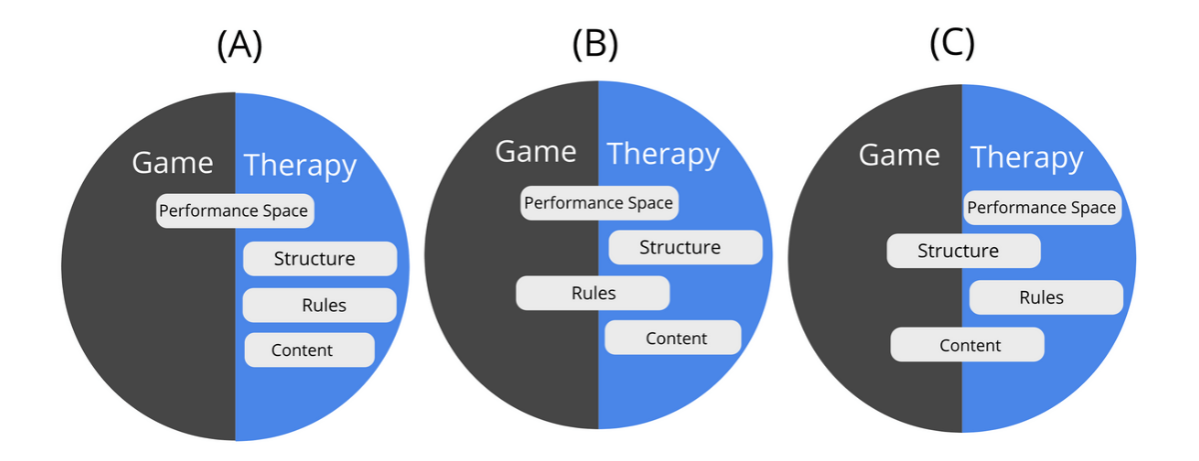
Three examples of strategies to integrate game worlds from existing therapeutic activities: (A) Creating a reward system based on the results. (B) Restructuring the rules and performance space. (C) Restructuring the content and structure.

Based on game and therapy world components, we have identified 3 different strategies that have been generally used to create an integrated game world that overlaps with an existing therapeutic activity (Figure 2). One strategy, the *reward systems strategy* (Figure 2A), is built around enhancing motivation by creating a reward system based on *the performance* of users in the therapeutic activity. For instance, if social competition is thought to be particularly appealing toward a target audience, simple mechanics such as a “leader board” could be used to create a feeling of competition based on how well users perform in the therapeutic activity in relation to others (see [45]).

Alternatively, another strategy is to *restructure the rules and performance space* (Figure 2B) of the target therapeutic activity to provide a more engaging experience. For example, in our gamification of the goal setting activity used in cognitive behavioral therapy, we added a time constraint *rule* and wager-based mechanism to the *performance space* to provide users with an experience of challenge when completing their goals. Users would set a self-imposed time limit for each goal (with less time receiving more points) and place a wager to reflect how confident they are in their ability to accomplish the task [10]. In the third strategy (Figure 2C), *the content and structure within the therapeutic activity* could be enhanced through *a narrative guidance framework*. This example could be seen in a gamified therapeutic activity used in cognitive behavioral therapy for burnout [22]. In this example, clients are asked to choose positive thoughts in response to stress-inducing work scenarios. In the gamified version, the therapeutic process is presented through the narrative guidance of a fictional character, with metaphors such as monsters representing negative statements, which players need to “shoot down,” and the cognitive state of players represented as an overloaded file cabinet that players need to clean up and fill instead with compliments. The whole therapy procedure is segmented into different levels, with players being guided through the levels as they complete each aspect of the therapy (ie, first recognizing negative thoughts, then identifying alternative positive ones and finally practicing with real-life situations) [22].

Overall, integrating a game world directly into an existing therapeutic activity provides several distinct advantages. First, interactions within the game world can be linked more directly to the interactions within the therapeutic activity, thus making them more meaningful and relevant to the therapeutic outcome. This is opposed to a nonintegrated approach with a separate game world (eg, Figure 1), where core player actions in the game space (such as going through various scenarios to learn about the dangers of drugs) play a more supportive role toward the outcome of the therapeutic activities [17]. In the integrated game therapy world used in the burnout gamification for instance, the actions of players in the game world (shooting down negative statements to earn points) are shared with those of the therapy world (rejecting negative thoughts as part of the therapy process) [22]. In addition, as the integrated game world also contains many elements that are shared with an original therapeutic activity, the intrinsic motivation of clients within the therapy world would more likely remain in the integrated game world as well. As such, users are likely to ascribe more value toward their actions in the integrated game world, as they feel it would lead them to a serious therapeutic outcome that helps improve their well-being. This is opposed to the effectiveness of discrete game worlds, which often depend on the entertainment quality of the game world itself. However, there is also the risk that altering different aspects of the therapy when integrating it with the game world could (negatively) influence the potential value of the therapy. For example, in gamifying a cognitive bias modification (CBM) task used to treat alcohol addiction, the authors cautioned that adjustments made to the original CBM paradigm as part of the gamification process (eg, changing the way in which the stimuli is presented, ie, the speed or frequency in which the alcohol image is shown, to make the training more challenging or changing the control paradigm, ie, requiring users to use different keyboard buttons or control devices to respond to the stimulus) could risk making the original training ineffective [46]. Cognitive training tasks in this domain (such as the Go/No-go task) require the stimuli and feedback to be presented at a specific time interval (1500 and 500 ms, respectively) [43], and it is unclear whether changing this original mechanism (eg, shortening the response time to create different challenge levels) would impact the therapeutic effectiveness.

### Our Proposed Method: Designing Gamified Therapeutic Interventions Through a Dual-Loop Design Model

During the design of the gamifications, we noticed how the core interaction process within a therapeutic activity shares many similarities with the concept of the core-game loop [47]. In game design, the core-game loop represents the fundamental action–feedback loop of a game, where players complete a specific task fulfilling an in-game objective and receive feedback based on their action. Players then use this feedback to improve their understanding of the game rules, update their strategies, and move on to complete the next task. The process then repeats itself throughout the game with the tasks becoming progressively difficult. Similarly, a therapeutic intervention or activity could also be viewed as taking place within a recurrent loop (ie, the therapy activity loop), as it generally consists of repeated tasks (although with different content) that clients must undertake to achieve the cognitive or behavioral change that leads to the desired health goal. After completing a task they would receive feedback on their performance and progress onto the next task with a different content but governed by the same rules and principles.

This observation led us to conceptualize the Dual-Loop Design model in which we propose that a core-game loop could be designed around the therapeutic activity loop, thereby enhancing the user experience of each of the therapy components. An earlier example of a gamified therapeutic activity being designed around this concept can be found in our previous studies, where we designed a goal setting gamification around the core-game loop of selecting different tasks leading to desirable therapeutic outcomes [10]. Based on our Dual-Loop Design model, the 3 components of a therapeutic activity which form the main part of a therapeutic loop are presented in Textbox 1.

**Components of a therapeutic activity.**

**Sample**

The sample refers to a specific task presented to the client, which is drawn from a list of possible tasks. For example, in the cue-specific response inhibition training task [43], clients are presented with a specific alcohol stimulus image (an image of a full or empty bottle of beer, etc.) which they would need to respond to.

**User action**

The user actions refer to the actions required of clients after being presented with the sample. This could occur both in the digital domain and in the physical world. For example, in the cue-specific response inhibition training task used in the treatment of alcohol addiction, clients can either choose to respond to the image of the stimulus in the task by pressing a button on the keyboard or choose not to respond by withholding their action.

**Therapy principle**

The principles refer to the logic within the therapeutic task that determines whether the actions of the user constitute a correct or appropriate response, which when carried out should progressively lead to the desired positive cognitive or behavioral change. For example, in the cue-specific response inhibition training task, clients must respond by making a correct action based on the stimulus presented within a specific time limit to be successful (ie, the desired response would be to press a key on the keyboard when shown a nonalcoholic image and withhold their response when an alcoholic image appears and letting it disappear).

**Figure 3:**
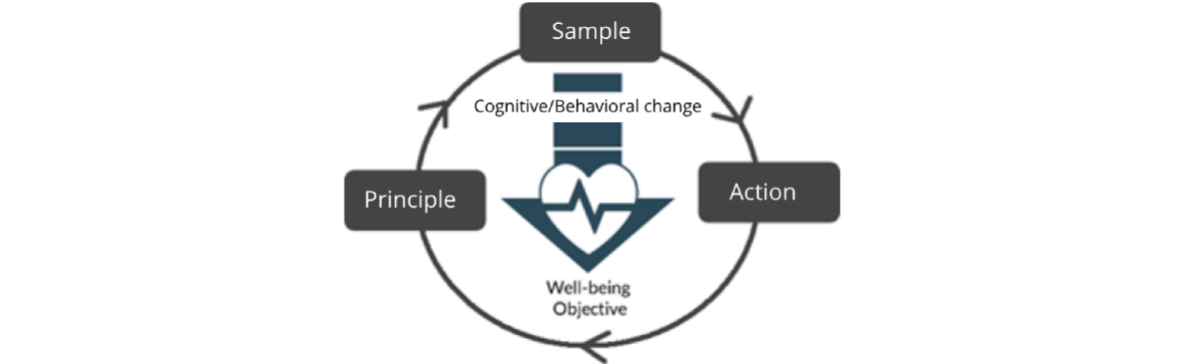
The therapeutic activity loop.

These components as well as the intended cognitive/behavioral change and the overall well-being/health objective are what constitutes the therapeutic activity loop (Figure 3). More specifically, in each iteration of the therapeutic activity loop, the client is given a *sample* task. They would attempt to carry out the *actions* required to complete the task based on the governing *therapy principles*. After completing the task, the client is then presented with the next sample task to complete and the process would repeat itself. The tasks presented to clients could cascade in difficulty or be used to represent a wide range of situations in the therapy (eg, in the CBM training for alcohol addiction, clients are presented with stimuli depicting different types of alcoholic beverages to better cover the large variety of alcohol drinks they may encounter in daily life [44]). By repeating this process, clients are able to change their cognitive state or behavior in a way which leads to a better health outcome.

**Figure 4:**
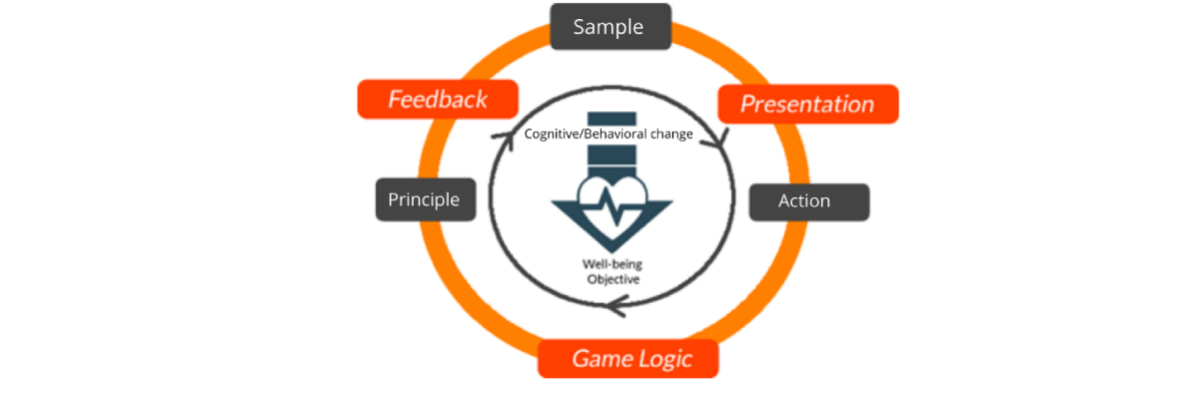
A core-game loop encompassing the therapeutic activity loop.

The main gamification process starts by formulating a core-game loop upon the therapeutic activity loop. More specifically, the gamification designer decides on the following 3 aspects (Figure 4).

- Can “presentation design” make the therapy more “gamey” and motivating and what are the implications toward the existing “sample” and “actions” of the therapy? For example, when a narrative metaphor is used to represent negative thoughts or maladaptive characteristic traits as enemy creatures [29], would this make the “sample” and “action” (eg, the task of recording the various triggers of addiction) more interesting?
- Can “game logic design” make the therapy more fun and what are the implications toward the existing “action” and “principles” of the therapy? For example, in our gamification of the approach bias training for alcohol addiction, we added a game logic in which players would need to push alcoholic and pull nonalcoholic pictures based on the beat of the music, instead of at a set time interval as in the original version of the training. This was done to enhance the underlying “action” (ie, the task of pushing away alcohol-related stimuli and pulling closer nonalcohol-related stimuli) and “principles” (ie, players must complete the tasks correctly within a set time limit) of the original therapy.
- Can “feedback design” make the therapy more fun and what are the implications toward the existing “principles” and “sample” of the therapy? For example, when a chain bonus system is added to the cue-specific response inhibition task (ie, users get an increasing number of points for consecutive correct actions, which resets when they make a mistake), would this influence the “principles” and “sample” of the therapy in a positive manner? More specifically, when players are presented with the next “sample” (ie, the task to inhibit their response toward an alcohol-related stimulus), would they put more effort in avoiding mistakes, as they would lose their chain bonus?

After deciding on the 3 aspects, the designer could then choose to add structural game elements to further enhance the core-game loop experience and encourage sustained engagement across the loops [48]. For instance, a “level progression system” could be added where players would be provided with experience points upon successful accomplishment of each task in the gameplay loop, which they then could use to “level up” their in-game characters, providing them with a sense of progression. Players could also be ranked based on their performance on a “leaderboard” to create a sense of competition. Another example is a “game-level system,” where completion of the tasks could be used to unlock different portions of the narrative experience, encouraging players to complete more tasks in the loop to complete the story.

**Figure 5:**
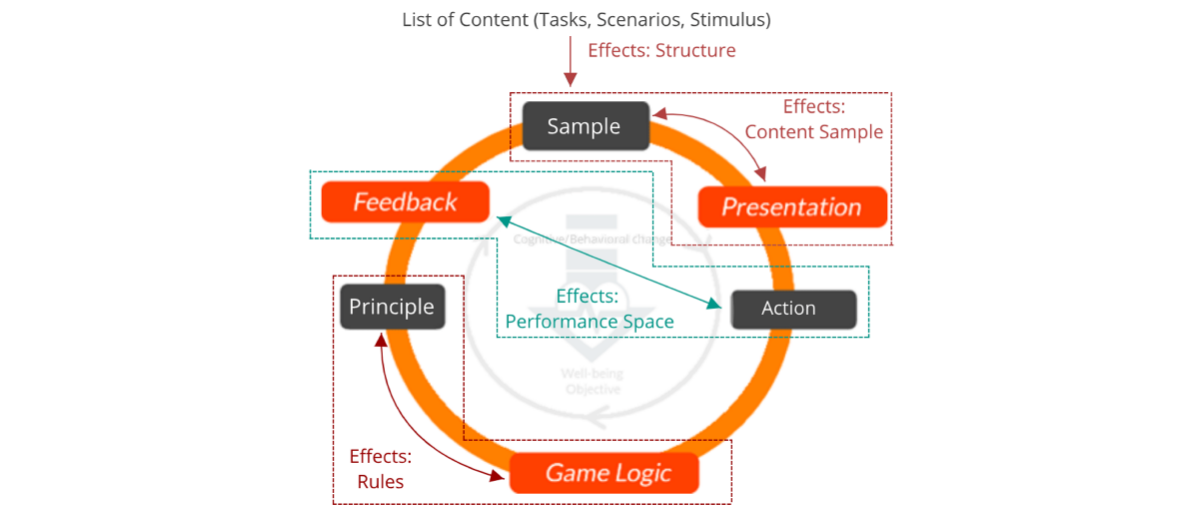
Effect of the core-game loop on the therapy game world.

When viewed from the Dual-Loop Design model, the components in the therapy world concept discussed in the “Strategies for Creating an Integrated Therapy and Game World” section serve to highlight how the different aspects of the original therapy could be affected through the gamification design process. Figure 5 provides a summary of the relationship between the therapy game world model and the dual-core–game loop. More specifically, the “performance space” in the therapy is affected mainly by the “feedback” design choice and the “user action” within the original therapeutic activity loop. The “rules” of the therapy are affected mainly by the “game logic” design choice and the “therapy principles” within the original therapeutic activity loop. The “content sample” is affected mainly by the “presentation” design choice and the “sample” within the original therapeutic activity loop. Finally, the structure of the game and therapy world is influenced mainly by the structural game elements, which the designers choose to use in their game design as this affects the nature of how each sample in the loop is drawn and presented to players (eg, a game-level system could be implemented which would mean that in each game loop, the sample that users interact with would be progressively more challenging). To balance the game and therapy world experience, players would need to take into account how their choices in designing the core-game loop influence the elements of the therapy game world.

### The Case Studies

#### Designing of The Case Study Gamifications

To provide more knowledge regarding the principles that can be used in the gamification of mental health care treatments, we designed and developed 4 different gamifications of existing therapeutic interventions. These gamifications are presented as case studies in the manuscript to show the result of how our proposed model could be used in the practical design and analysis of gamifications for mental health care. The gamifications were designed in collaboration with experts from serious games design as well as researchers and clinicians in the domain of psychological interventions. To ensure the generic applicability of the principles, the gamifications covered 2 main classes of intervention types (ie, cognitive training and cognitive behavioral therapy) and 2 main categories of mental disorders (ie, externalizing and internalizing disorders). In addition, 2 of the gamifications were of computerized cognitive training modules that are mostly implemented without the interference of a therapist, and the other 2 were designed to be used as part of a blended therapy program through face-to-face sessions with a therapist. The 4 gamifications include the ReadySetGoals mobile app (*cognitive behavioral therapy, blended therapy, externalizing disorder*), the Addiction Beater computer application (*cognitive training, computer based, externalizing disorder*), the Zen Garden mobile app (*cognitive behavioral therapy, blended therapy, internalization disorder*), and the Één klein probleempje (Small problems) computer application (*cognitive training, computer based, internalizing disorder*).

#### The ReadySetGoals Design

##### Purpose

**Figure 6:**
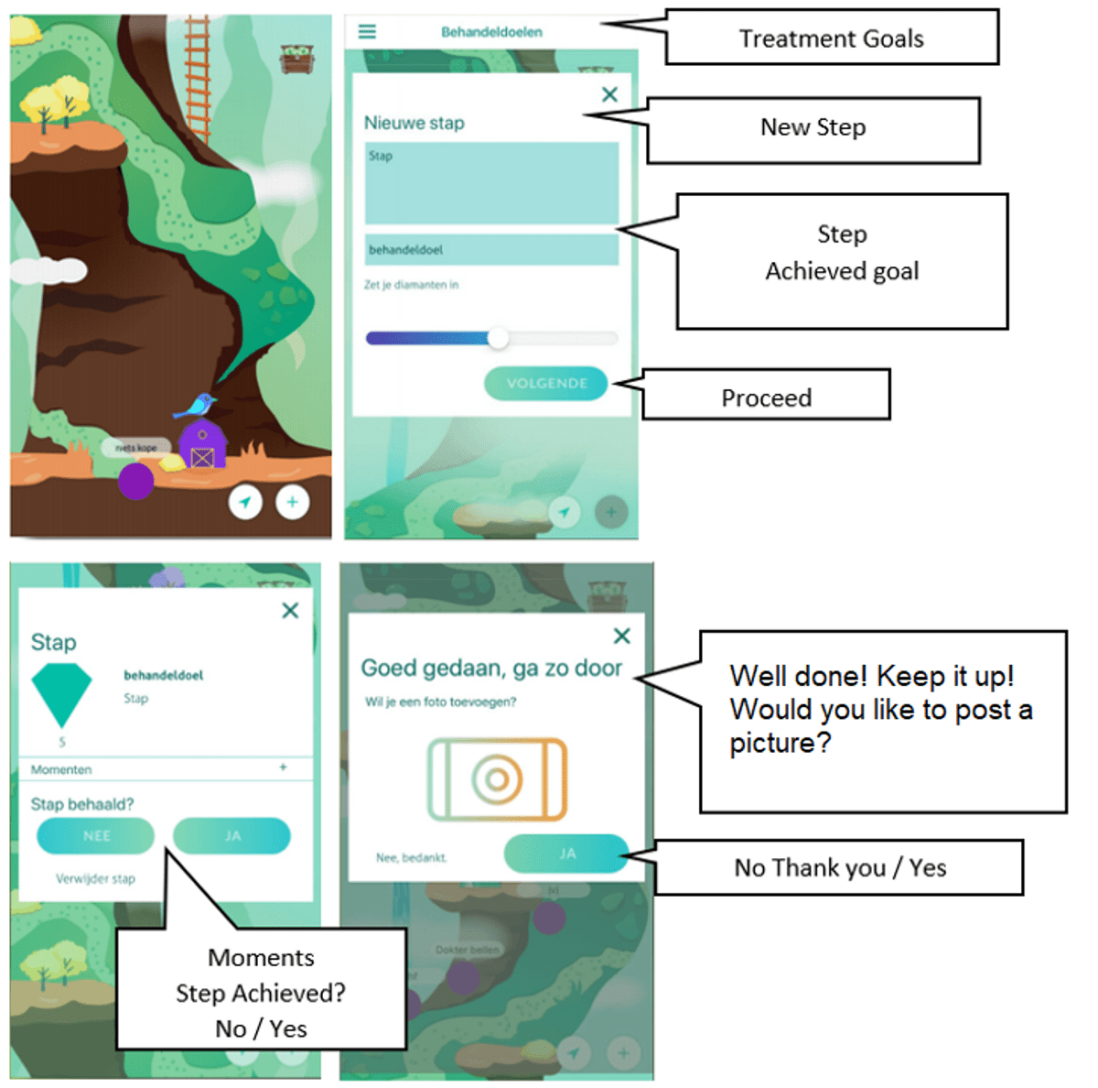
Screenshots of the ReadySetGoals gamification.

The ReadySetGoals is a gamification of the goal setting activity commonly used as part of a treatment plan at the beginning of therapy in mental health care. In addiction treatment, this activity plays a key role in addressing not only substance use problems, but also anxiety and depression symptoms. The goals tend to focus on encouraging clients to change their typical behaviors or aspects of daily life, in order to improve mood and decrease substance consumption. However, it is generally difficult for them to adhere to such goals and clients tend to experience difficulty in putting therapeutic insights into practice. Therefore, the ReadySetGoals was created as a gamified mobile app based on a risk-taking mechanism to encourage users to set and complete goals that are beneficial to their treatment. Players set goals to achieve various therapy-related tasks and then place a wager on how likely they feel they are able to achieve those goals. Structural game elements such as a progression-based reward system (eg, users would progress along a path for each goal set) were implemented. As part of a blended therapy program, the app was used during the face-to-face session between the client and the therapists. During each session, the client would decide together with the therapist on what long-term goals and tasks would be appropriate. In addition, the level of difficulty (and thus the reward which would be received) of each task was decided in collaboration with the therapist. In the following session, the therapist would review the tasks and credit points to the client for each task that was completed satisfactorily, evaluate the overall progress of the long-term goals, and set new tasks for the following week through the ReadySetGoals app. Figure 6 shows screenshots of the gamification (see [10] for in-depth details of the gamification design process).

##### The Gamification Strategy and Dual-Loop Design of the ReadySetGoals App

**Figure 7:**
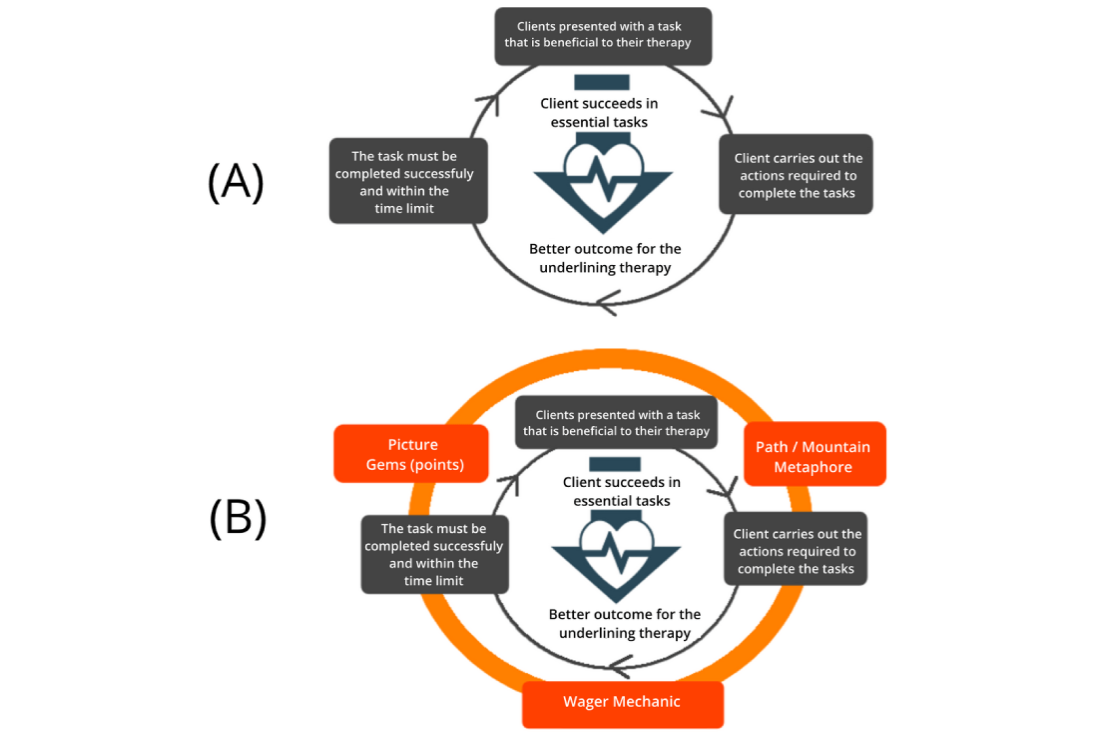
The core-game loop and gamification design of ReadysetGoals.

In the gamification of the ReadySetGoals app, the desired behavioral change was defined as *encouraging users to persist and succeed in tasks that are essential in therapy*. Users are encouraged to set goals to complete the various therapeutic activities that are given to them in different domains (eg, find and carry out alternative rewarding activities such as pleasant hobbies, as a way to become less dependent on drugs). In the original therapeutic goal setting activity, clients discuss with the therapist and decide on a goal which they would need to complete to achieve a better outcome in their treatment. They are then required to break down the goal into smaller tasks (*sample*), decide on a time limit, and then carry out the actions required to complete the tasks (*action*). Clients are successful if they complete the tasks within the agreed time limit (*principles*; see Figure 7A).

To determine what game elements could be used to increase user engagement in the therapy loop, we carried out multiple sessions including a discussion session; brainstorming session; and survey study with therapists, care managers, domain experts, and clients of an addiction center (see [10]). The results showed that personality traits such as sensation seeking and impulsivity are very prevalent among clients with addiction problems [49] and thus we determined that a risk-taking game mechanic would be appropriate in engaging these clients. Therefore, we decided to add a wager rule as the main game mechanic in the gamification (*game logic*). Based on this mechanic, players receive points (in the form of diamonds) that they could use to “bet” on themselves based on how likely they feel they can complete a specific task. Players are then rewarded in proportion to the risk taken (the amount of wager placed and time limit they set themselves). In addition, we represented the small steps that users are taking to progress within the therapy through the metaphor of climbing a mountain (*presentation*). As users complete tasks, they move further up the mountain until they reach the destination on the top. Users are rewarded with points for successfully completing tasks and they are also able to view pictures taken as proof when they complete their tasks on the mountain (*feedback*; Figure 7B).

##### The Therapy Game World of the ReadySetGoals App

**Figure 8:**
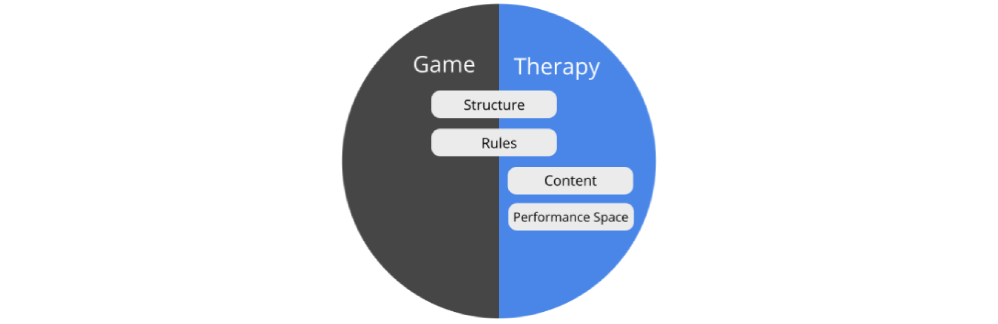
The therapy game world of the ReadySetGoals gamification.

When designing the ReadySetGoals gamification, we determined that the *structure* and *rules* of the therapy were flexible enough to be adapted without severely impacting the therapeutic effect. By using the mountain metaphor and presenting the smaller steps to obtain the goals on a progression map, we restructured the way tasks are presented to users. For example, in the initial version of ReadySetGoals, users need to complete easy tasks at the beginning before moving on to more difficult ones and they were limited in the number of tasks they could challenge themselves at one time. Adding the wager mechanic also created a rule where players are rewarded more for achieving tasks in a shorter period, thus changing the original rule where players are provided with a fixed deadline. We expected the design of the gamification to have a minimal impact on the original *content* and *performance space* of the therapy (Figure 8). The goals that users need to complete were the same as those given in the original therapeutic setting and the actions that users would need to take also stayed the same. Similar to the original activity, the therapist and client would collaborate together to set the long-term goals and task difficulty during each therapy session (eg, the therapist and client would first select the relevant area in life which they would like to work on and set the goals and tasks together). The client would also set a specific date and time deadline they feel they could accomplish the task and in the following week, the therapist would review the progress of the goals and tasks and provide feedback to the client.

#### The Addiction Beater

##### Purpose

**Figure 9:**
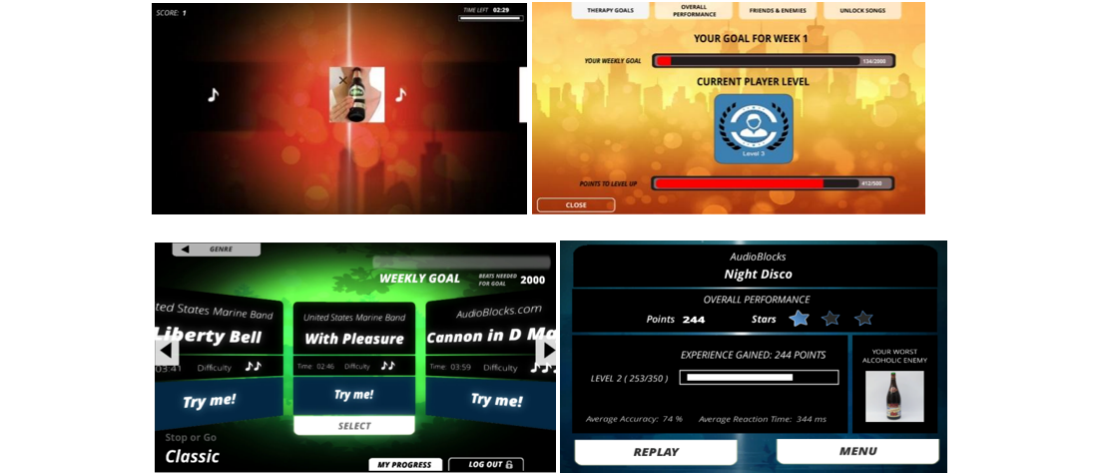
Screenshots of the Addiction Beater game.

The Addiction Beater is a gamification of the therapeutic activities included in different CBM training modules based on a music rhythm game concept and was designed to support the treatment of alcohol addiction. Such CBM modules have been used in digital cognitive training programs as an add-on therapy for addiction disorders, by helping clients to retrain maladaptive cognitive processes in substance abuse (see [50,51]). In the original training, people’s motivation to engage and persist in the training was quite low due to the repetitive nature of the task. To address this problem, the Addiction Beater gamification was developed based on a music rhythm game concept to provide a more engaging training experience. Two modules used in CBM training were gamified: the cue-specific response inhibition training and the approach bias training. In the gamification, users are challenged to respond to the alcoholic stimulus based on the beat of the music. For instance, in the approach bias training module, users are challenged to “push away” images of alcoholic drinks using the “up” arrow key and “pull closer” images of nonalcoholic drinks using the “down” arrow key on their keyboard based on the musical beat of different songs. When carrying out the training tasks, users can select from a list of different songs to use as part of their training. The current version has over 20 songs in more than 4 different genres (Rock, Electronic, Classic, International, etc.). As users continue to train, they would be able to gain experience points, which will unlock more difficult songs (Figure 9).

##### The Gamification Strategy and Core-Game Loop Design of the Addiction Beater

**Figure 10:**
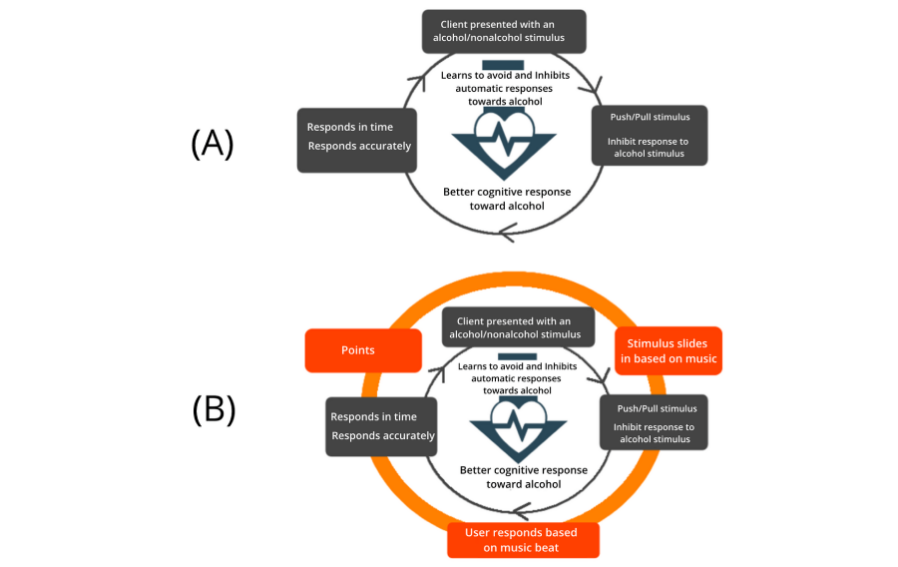
The core-game loop and gamification design of Addiction Beater.

When viewed from the Dual-Loop Design model, in the original cognitive training clients are presented with an image of either an alcoholic or nonalcoholic beverage (*sample*). In the cue-specific response inhibition training task for example, clients need to withhold their response when an image of an alcoholic beverage is presented and respond when the image displays nonalcoholic beverages by pressing the spacebar key (*action*). In both training tasks, clients would need to respond with the correct action based on the image shown within a specific time limit to be successful (*principle*). The repeated actions of clients in both training tasks enable them to learn to inhibit their automatic responses toward alcohol and reduce their automatic tendency to approach alcohol, which in turn allow them to better respond toward alcohol-related cues in real life (Figure 10A).

The gamification process for the Addiction Beater started with an initial analysis of the digital training modules. Initial brainstorming sessions were carried out to generate ideas on how the original *sample*, *action*, and *principle* of the cognitive training could be enhanced through gamification. Through a discussion with addiction researchers and clinical experts, different concepts were generated and refined into playable prototypes. In the concept that was later selected by the domain experts, the alcohol-related and nonalcohol-related images were presented to players based on the beat of the music. A beat detection algorithm was developed to ensure that the images would slide into the center of the screen based on the beat of the songs (*presentation*). Players would then need to respond in a timely and correct manner based on the rhythm of the music (similar to rhythm games where players tap keys or hit drums during different musical beats in the song), according to the original therapeutic principles (*game logic*). Players would receive points for each correct response, which was afterward upgraded into a “combo system” in which players would receive an increasing amount of bonus points for providing multiple correct response consecutively (+1 for the second correct response, +2 for the third correct response, etc.; *feedback*). Afterward, the feasibility of our music rhythm game concept was further examined in a preliminary evaluation session with heavy drinkers of different age groups. The results of the evaluation prompted us to add structural game elements, such as a level progression system in which players would earn experience points and level up for each training round completed. In addition, as the target audience of this gamification were people who were suffering from alcohol addiction, we avoided taking design choices (such as adding structural game play elements) that would result in our gamification sharing key characteristics found in addictive games (eg, 24-hour online games with an extensive in-game social network footprint, containing role-playing elements that strongly appeal to the sense of escapism) [52]. Furthermore, when implementing the gamification, we ensured that Addiction Beater was played under the supervision of the therapy staff, thus making it difficult for excessive gameplay.

##### The Therapy Game World of Addiction Beater

**Figure 11:**
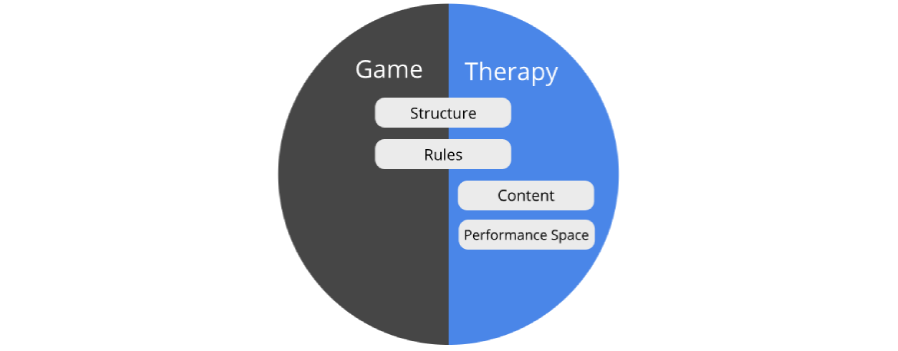
Therapy game world analysis of the Addiction Beater Gamification.

Early discussions carried out during the design of the CBM gamification revealed concerns about whether modifying the *performance space* and *content* of the original training modules might reduce the therapeutic effectiveness (Figure 11). Therefore, these 2 elements were kept constant in the gamification. The type and number of alcohol-related and nonalcohol-related images shown to the players (*content*), the actions players need to perform (pushing away alcohol-related images and pulling in nonalcohol-related images), and the feedback received (text feedback indicating they responded correctly or not; *performance space*) were kept the same as in the original training. While we kept most of the *principles* the same (eg, the time given to users to respond to each stimulus), we added an additional rule that players would need to time their response based on the beat of the music as a way to provide players with an experience of challenge and immersion. Finally, the *structure* of the therapy was modified in that the number of stimuli presented to the users in each training round was based on the length of the song played (and not based on a fixed amount as in the original therapy).

#### The Zen Garden App

##### Purpose

**Figure 12:**
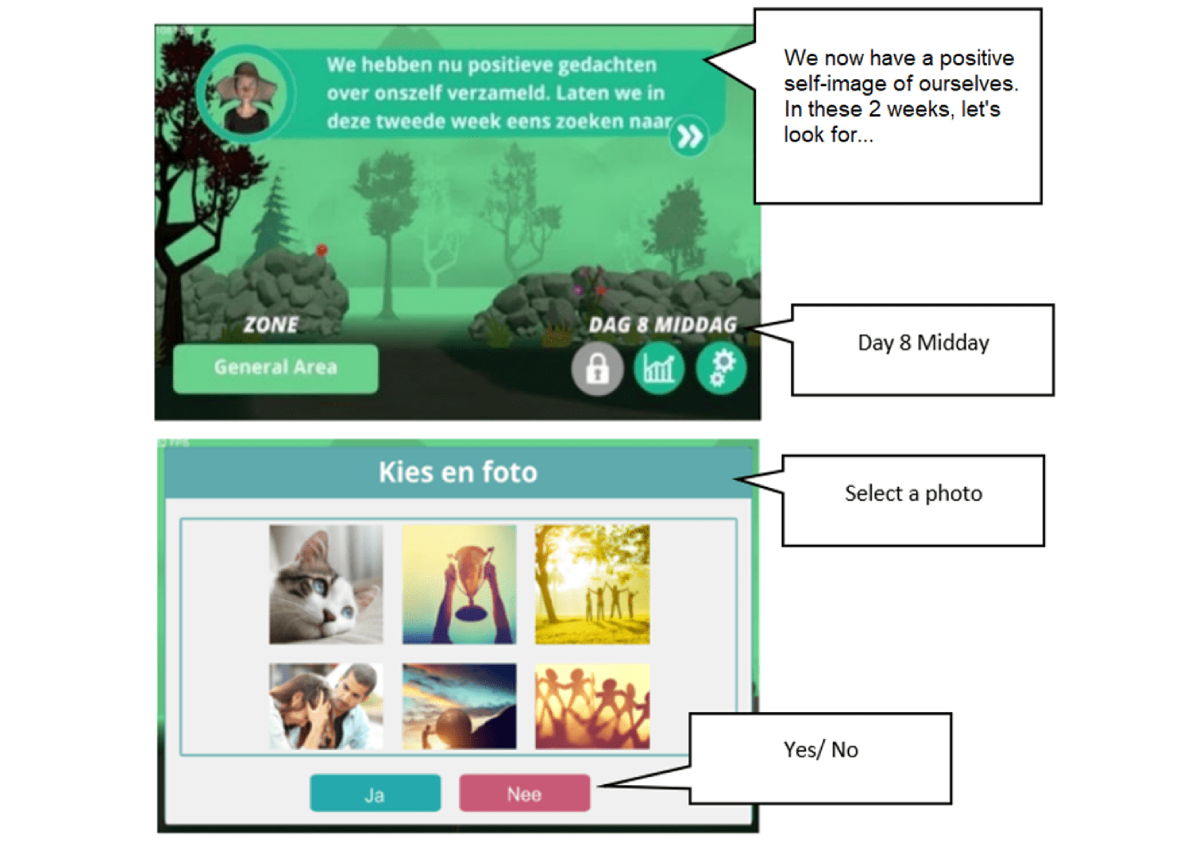
Screenshots of the Zen Garden.

The Zen Garden is a gamification of the Competitive Memory Training (COMET) therapy used in the treatment of low self-esteem problems [53]. Low self-esteem has been found to be a factor in the development of a range of disorders, such as anxiety, depression, and eating disorders [54], leading also to self-harming and suicidal behaviors. The COMET therapy was developed to teach clients to retrieve positive and functional self-referent instead of negative information in situations that normally trigger negative thoughts and emotions linked to low self-esteem. The current Zen Garden was developed based on this therapy as a mobile app and adopts the aspects of playful interaction and progression. In this gamification, users are encouraged to plant, grow, and collect positive self-referent resources of themselves (eg, stories, photos, songs) and revisit the plants to strengthen memories of their positive qualities (Figure 12).

##### The Gamification Strategy and Core-Game Loop Design of the Zen Garden

In the original therapy, clients are asked to adjust dysfunctional negative self-views that impact their self-esteem (eg, clients thinking “I am worthless”; *sample*). They first identify positive personal characteristics that are inconsistent with their negative self-images (being honest, helpful, etc.) and describe examples of actions or situations in their daily life that are illustrative for those characteristics (eg, I helped a friend yesterday). These new positive characteristics of themselves are made more emotionally salient by imagining them repeatedly. In a step-by-step fashion, these positive images are further supported by adding a self-confident body posture and facial expression, positive self-statements, and self-selected positive music (*action*). Overall, the COMET training is congruent with Brewin’s [55] competitive memory retrieval account of the working mechanism of cognitive behavioral therapy [55]. By making functional positive self-opinions more emotionally salient, clients will be able to better retrieve them from long-term memory, overruling the retrievability of the original dysfunctional negative self-opinions (*principles*), which would result in the improvement of self-esteem.

**Figure 13:**
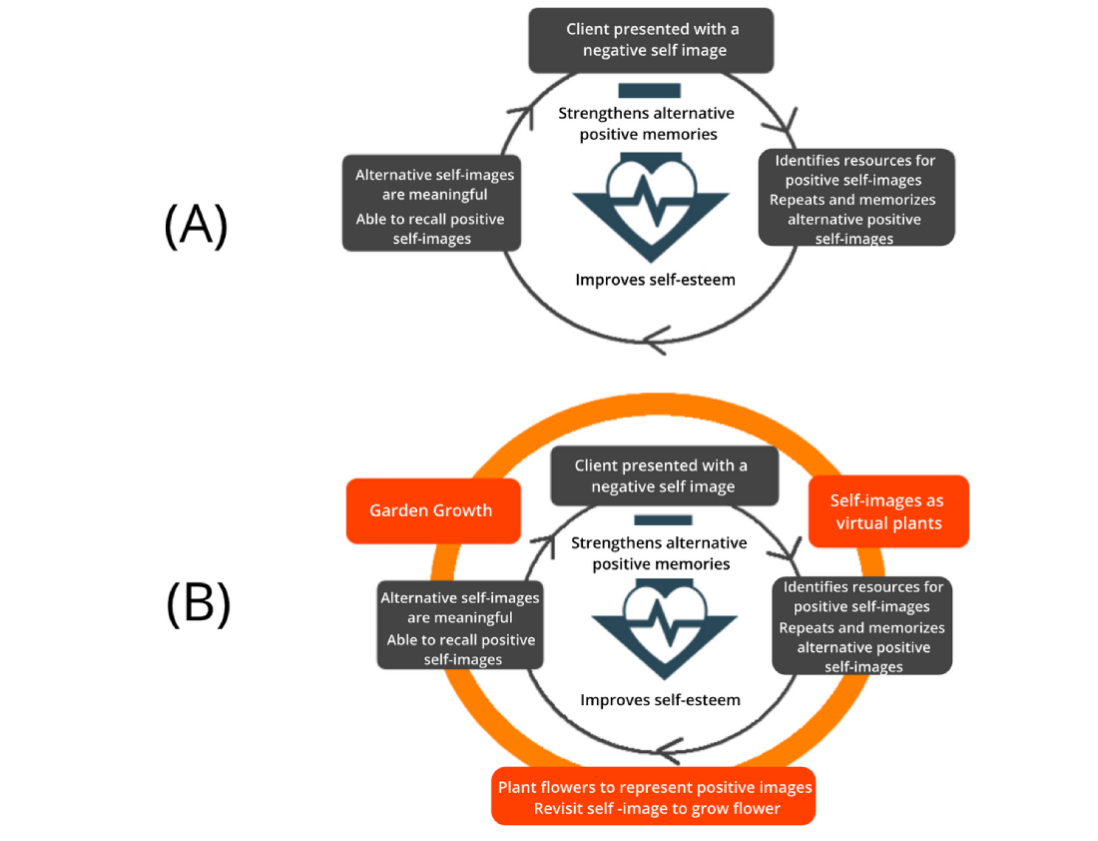
The core-game loop and gamification design of Comet Garden.

During the gamification of the COMET therapy, early game concept testing with a group of clients who completed the original therapy revealed that they tended to react negatively to game experiences related to challenge and competence, as failure in game tasks tended to reinforce the negative self-belief about themselves instead of motivating them to retry and complete the task. Therefore, in the gamification of the COMET therapy, we focused on more passive playful experiences, such as playful interaction and narration. The rationale for this design was to provide players with a sense of achievement without evoking fear of failure. We eventually adopted the concept of a virtual garden, deployed on a mobile device, where counteracting positive characteristics are represented as virtual plants in a 3D garden. The visual style of the garden was based on a peaceful Zen garden concept, with nature sounds such as that of water flowing incorporated into the garden to provide players with a feeling of serenity to help reduce anxiety (*presentation*). The garden is divided into different areas, each representing a negative self-belief, and users plant and grow flowers in each area by adding positive information about themselves. Text-based stories, images, and music representing real-life examples of those positive beliefs are added to each plant to help them grow (*game logic*). As users add more positive beliefs, the garden becomes more visually beautiful (*feedback*). Figure 13 summarizes the core-game loop and gamification design of the COMET application.

##### The Therapy Game World of the Zen Garden App

**Figure 14:**
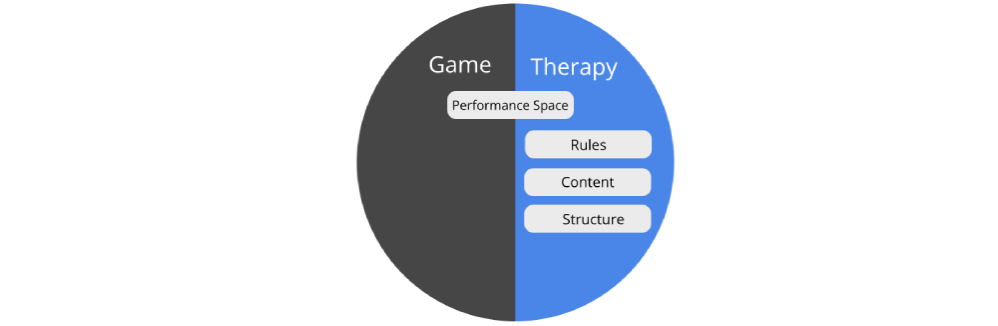
Therapy game world analysis of the Zen Garden gamification.

The gamification process of the COMET therapy relies on elements of playful interaction and the feeling of achievement from collecting and growing positive memories. The *rules*, *content*, and *structure* of the original therapy remain largely unchanged (Figure 14). Similar to the original therapy, players are asked to identify negative self-beliefs and then come up with alternative positive representations about themselves. Afterward, they add evidence for such new self-beliefs in their daily life and reflect on such evidence to strengthen their alternative positive self-beliefs (*rules and content*). The task given to participants each week was consistent with the original therapy (*structure*). More specifically, when used as part of a blended therapy activity, players would collaborate with the therapist during the first week to identify negative self-images, plant their first flower (representing a contradicting positive self-image/personal characteristics), and add self-referencing stories. In the following 4 weeks, players would add more stories (week 2), photos (week 3), and songs (week 4), which represent real-life examples that contradict their negative self-belief and review them daily through the Zen Garden app (similar assignments are given to clients during each week in the original therapy). Afterward, clients would share their garden with the therapist who would provide feedback about the evidence added and later clients would further review their contradicting positive self-images to strengthen them (week 5). However, due to the garden metaphor and technological limitations, the amount of resources that could be added for each flower was fixed (up to a maximum of 5), limiting the number of positive self-beliefs players could add, thus impacting the *performance space* of the therapy.

#### Één Klein Probleempje (A Tiny Problem): Social Anxiety Gamification

##### Purpose

**Figure 15:**
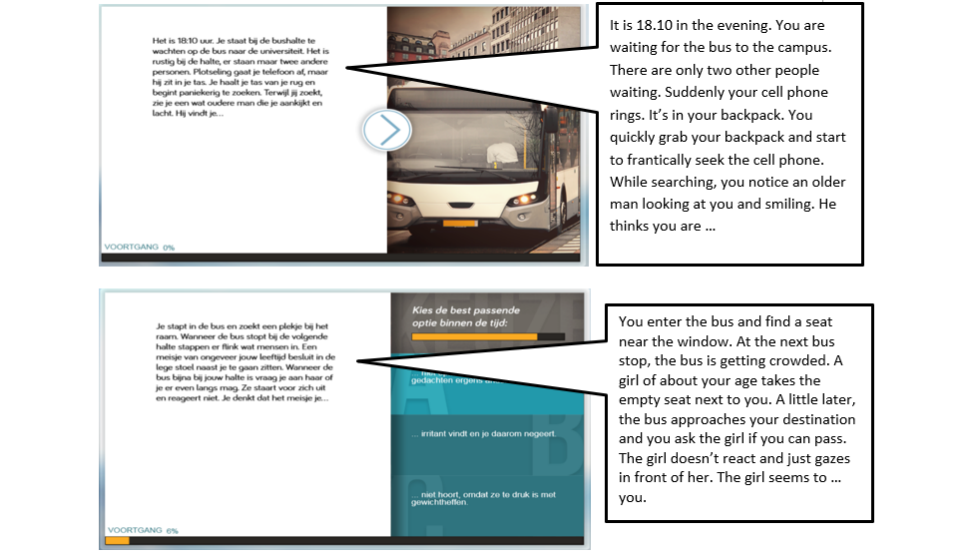
Screenshots of the “A Tiny Problem” gamification.

The Één klein probleempje app is a narrative-based gamification of the cognitive bias modification of interpretations (CBM-I) training to address anxiety problems [56]. People with anxiety problems are generally inclined to interpret ambiguous information in a negative and threatening way, which in turn reinforces and exacerbates their anxiety symptoms. Interpretation bias training interventions have been designed to change this maladaptive information processing mechanism by training individuals to make more benign interpretations of ambiguous situations. However, this type of intervention is generally perceived as repetitive and tedious as clients are requested to complete multiple training sessions [57]. The Één klein probleempje was developed as a gamification of the CBM-I training by adopting the element of narration to enhance training. In this gamification, the fragmented scenarios in the original training are transformed into an engaging and connected story. Branching sections were added to the story which allowed users to decide on how the story unfolds. As users navigate through the story, they are encouraged to interpret the various ambiguous situations in a more positive way. Figure 15 shows a screenshot of the gamification.

##### The Gamification Strategy and Core-Game Loop Design of the Één Klein Probleempje

**Figure 16:**
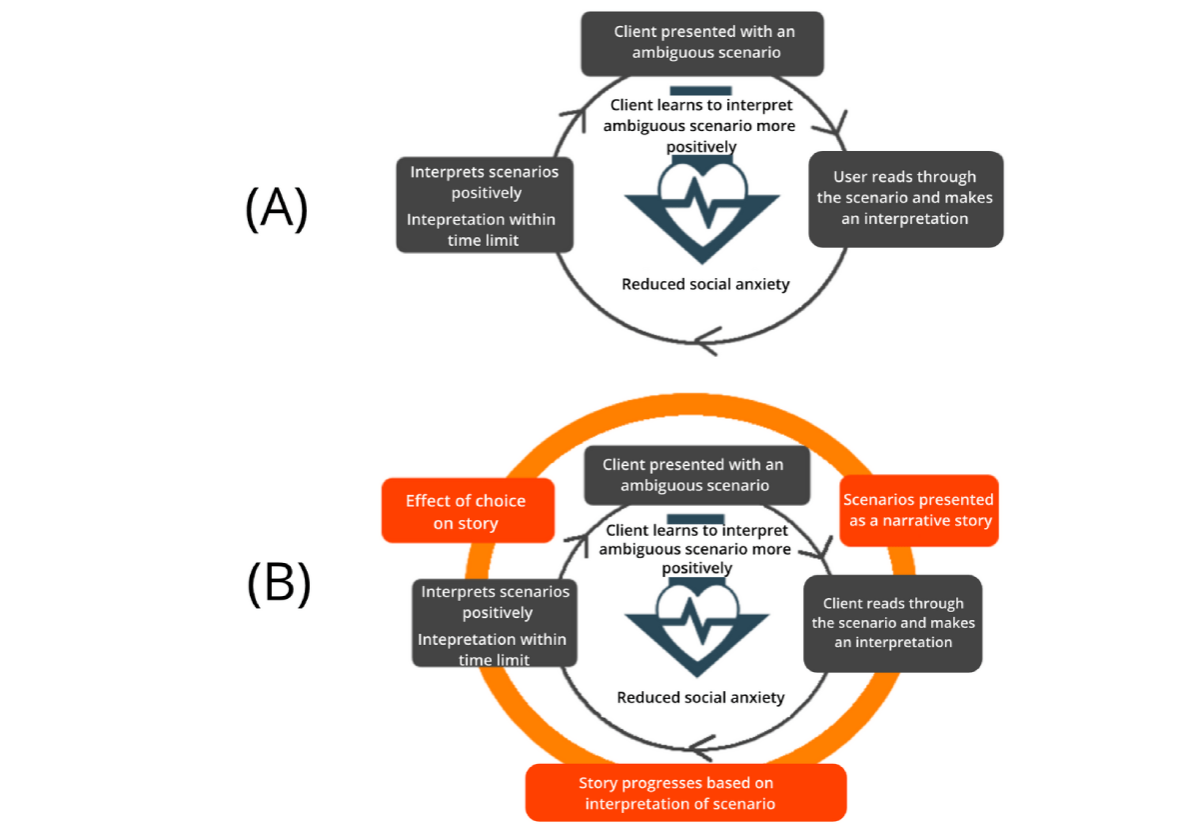
The core-game loop and gamification design of the small problems.

In the original CBM-I training, clients are first presented with an ambiguous scenario ending with a word fragment, which can be solved with either a positive or negative outcome. Clients need to read the text and resolve the word fragment in a meaningful fashion (*action*). An example scenario is the following: *You’ve finished writing the answer to the second question in your exam. You take a small break, looking at what’s left. You then realize that the questions left are more difficult than you had anticipated. Checking the watch, you decide you’ve planned your time w_ll [well].* A subsequent question relating to the interpretation (eg, *Will you have time to complete the exam?*) is then presented, and users need to provide an answer (*yes* or *no*; *Scenario*). To be successful, the client would need to resolve the word fragment and related scenario in a positive manner and within a time limit (*rules*) [58]. Repeated training helps clients learn to interpret ambiguous situations in daily life in a more benevolent manner, thus breaking the dysfunctional thinking patterns underlying anxiety (Figure 16A).

Because of the key role of text-based scenarios in the original training, we decided to use the element of narration to gamify the CBM-I training task. The different scenarios in the original therapy were tied together and presented to users as an adventure story (*presentation*). The aim was to engross users through the narration of the story, as they would be motivated to complete the training task and resolve ambiguous situations to find out how the story ends. To progress along the story, users need to provide a positive interpretation of each story scenario by selecting the correct option from a multiple choice list presented in a timely manner (*game logic*). A negative interpretation will lead users back to the same choice point and users would need to make the correct choice (ie, the positive interpretation) to continue with the story (*feedback*; Figure 16B).

##### The Therapy Game World of Één Klein Probleempje

**Figure 17:**
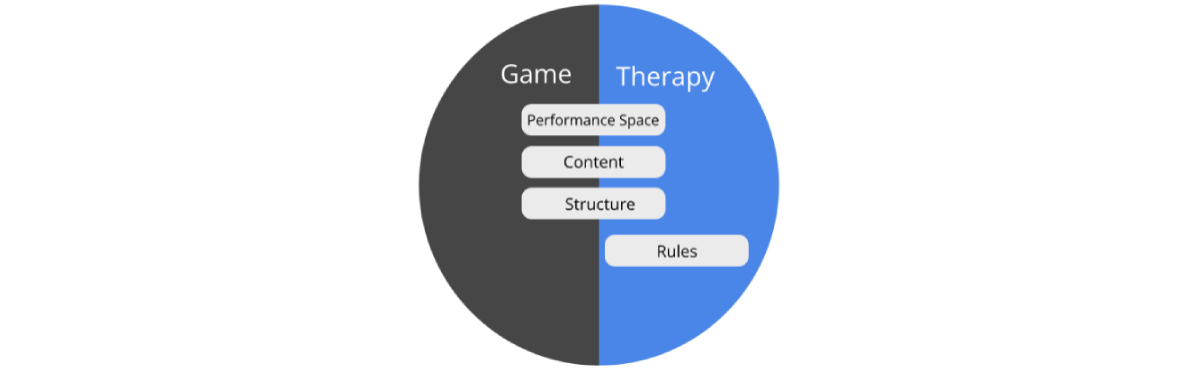
Therapy game world analysis of the Small Problems gamification.

In Één klein probleempje, the scenarios from the original CBM-I training were converted into a storyline, thus changing the original *content* of the training. In addition, the *structure* of the original training was modified (Figure 17). Instead of each scenario being presented in a random, unrelated order, they are presented in a logical order based on how the story unfolds. Through discussions with cognitive training experts, we decided that the content and structure of the scenarios were flexible enough to be adapted without severely impacting the effectiveness of the targeted training. In addition, instead of filling in a word fragment to complete the scenario, users would choose how the scenario unfolds by selecting 1 of 4 optional outcomes. This was done to provide a more natural user experience as users go through the story, thus impacting the *performance space* of the original therapy. The overall *rules* of the therapy were kept the same, as users are still forced to choose a positive outcome for the scenario to succeed in the therapy.

Table 2 provides a summary of the therapy loop of the 4 gamification case studies and Table 3 provides a summary of the overall gamification design of the 4 case studies.

**Table 2:** A summary of the therapy loop of the four gamification case studies.

Gamification	Existing therapeutic activity	Challenge of existing activity	Therapeutic activity loop				Desired specific behavioral or cognitive change		Desired overall well-being objective
			Sample	Action	Principles				
ReadySetGoals	Goal setting within cognitive behavioral therapy.	It is difficult to motivate clients to set and accomplish goals related to cognitive behavioral therapy.	Users presented with a task beneficial to their therapy to carry out in daily practice.	Users carry out the actions required to complete the tasks.	The task must be completed successfully and within the set time limit.	*Behavioral change:* Users are able to set and accomplish goals that are beneficial to their therapy.		Accomplishing goals (abstinence goals, skill attainment goals, etc.) leads to better therapeutic outcome.	
Addiction Beater	Cognitive bias modification training used in alcohol addiction treatment.	High dropout rate. Boredom due to repetitiveness, particularly with youth in prevention studies.	Users are presented with target stimuli (alcohol related or nonalcohol related).	Users decide to “push”/“pull” or inhibit their response toward a target stimulus which is explicitly alcohol related or associated with alcohol.	Users must respond in time and accurately.	*Cognitive change:* Users learn to avoid and inhibit automatic responses toward alcohol.		Lower relapse rate or reduced alcohol use.	
Zen Garden	Competitive Memory Training used in the treatment of low self-esteem.	The e-training version has problems related to low retention.	Users identify a negative self-image.	Users identify alternative positive self-images and rehearses them.	Users must be able to recall positive self-images.	*Cognitive change:* Strengthens positive self-images as opposed to negative ones.		Improvements in self-esteem.	
Één klein probleempje	Cognitive bias modification of interpretations training for anxiety.	Boredom during training, repetitiveness, and fragmentation of scenarios.	Users presented with an ambiguous scenario.	Users read through the scenario and fill in a word fragment, which can end positively or negatively.	Users interpret the scenario positively and within the time limit.	*Cognitive change:* Learns to interpret ambiguous information in a benevolent manner.		Reduce social anxiety.	

**Table 3:** A summary of the overall gamification design of the 4 gamification case studies.

Gamification	Game concept	Core-game loop elements			Structural game elements
		Presentation design	Game logic design	Feedback design	
ReadySetGoals	A risk-taking concept where users place wagers on their set goals.	Goal setting is presented through the metaphor of climbing a mountain.	Players receive points proportionate to the risk taken (the amount of wager placed and the set time limit) and the difficulty of the task. Players progress further up the mountain for each accomplished goal.	Successful task completion and risk taking are rewarded with points. Pictures of accomplished tasks are placed on the mountain path to provide a sense of achievement.	Player progression (the number of points accumulated increases player levels); gameplay progression (easy goals are placed at the bottom of the mountain and more difficult goals are placed at the top).
Addiction Beater	A music rhythm game concept where users must respond based on the beat of the music.	Alcohol- and nonalcohol-related stimuli are presented based on the beat rhythm of the music.	Users have to react to the stimuli (to the content or other features) as close as possible on the beat of the music.	Users receive points for each correct response and more points for consecutive correct responses (combos).	Player progression (players accumulate experience points for each correct response and level up); gameplay progression (players unlock more difficult songs after completing easier ones); social competition (players see their performance [response time, accuracy] in relation to others).
Zen Garden	A playful Zen garden where users can plant positive memories into flowers.	Players’ “self” is represented through a garden metaphor: negative self-beliefs are represented with zones within the garden and positive self-images are symbolized through flowers planted in the garden.	Users need to plant flowers to grow their garden and their positive self-images.	The more flowers are planted, the more the garden grows.	Narrative guidance (an avatar is used to guide the participants throughout the therapy and give them tasks).
Één klein probleempje	An adventure story–based gamification.	Each ambiguous scenario is presented as an element of a larger narrative story.	Users interpret the ambiguous scenarios and the narrative story responds to the players’ interpretations.	The results of the player interpretations are shown through narrative feedback.	Story progression (the story progresses as players interpret the ambiguous scenarios).

## Discussion

Overall, the 4 case studies were presented to help illustrate how our proposed Dual-Loop model and the game therapy world concept could be put into practice when designing gamification for mental health care. In each of the gamifications, different design elements in the core-game loop (presentation, game logic, etc.) were selected to correspond with the inherent characteristics of the components in therapeutic activity as analyzed through the Dual-Game Loop model. The therapy game world was then used to analyze the impact of the selected game design elements on the underlining therapeutic activity. The key reflections and observations from our use of the framework to design the gamification in the case studies are highlighted in Textbox 2.

**Key reflections and observations.**

**Presentation design**

In gamifications designed based on computerized cognitive training therapies, such as Addiction Beater and Één klein probleempje, the presentation was generally designed based on the limitations and characteristic of the stimulus used in the original computerized training as they tended to be predetermined. For instance, in Addiction Beater, it was unclear whether changing the visual size or color in which the alcoholic/nonalcoholic images were presented would impact the therapy effectiveness [43] and thus the presentation was enhanced mainly through audio. In Één klein probleempje, a narrative element fitted well as this allowed the text-based scenarios to be presented in an engaging manner without severely effecting the original content of the scenarios used for the cognitive bias modification training. Such considerations to presentation design are reflected in cognitive training gamifications developed in previous studies as well [22,46]. For cognitive behavioral therapies that were deployed as blended therapies (The ReadySetGoals and the Zen Garden), however, the presentation tended to be more abstract and dependent on the user (ie, goals to set or positive self-images). In such cases, a metaphor system was used to highlight the achievements of the user in the therapeutic activity (ie, presenting completed goals as steps on a mountain and positive self-beliefs that users added as gardens), acting as a form of virtual trophy, a commonly used gamification strategy to reinforce progress or good behavior from users [59].

**Game logic design**

In the Addiction Beater and Één klein probleempje gamifications, the therapy principles generally contained user interaction rules which were fixed (ie, respond to the stimulus within 500 ms) [43,44] and it is unknown whether modifying them would have an impact on the therapeutic effectiveness. In such cases, similar to the presentation design, rather than risk changing the original principles in the therapy, we added a simple game play mechanism upon the original user action in the therapy to enhance the gamified user experience (ie, while users still had to respond within 500 ms to the stimulus, this was matched to the beat of the music in Addiction Beater to create challenge). For cognitive behavioral therapies deployed in a blended format, completely new game logic and interaction mechanism were devised (the wager mechanism for the ReadySetGoals, etc.). The rules involved in these therapies were often more subjective and flexible (ie, in the goal setting activity, users set their own deadline and the success and failure of the goal are decided in collaboration with the therapist), giving more freedom in the game logic design. In a sense, the game logic design constraints and strategies employed for these gamifications are similar to those found on other gamifications where user interaction is primarily driven by the behavior of users in the real world (such as those designed to encourage behavioral change [60] or facilitate the self-management of a disease [61]).

**Feedback**

The feedback was generally designed as a way to quantify the achievements of the users within the therapeutic activities, with points or experience points being awarded for performance in ReadySetGoals and Addiction Beater. This numerical quantification was necessary to allow challenge-based structural game element, such as competition or player progression, to be employed (which coincided well with gamification strategies designed to treat explicit mental health disorders such as addiction [62]). For gamifications designed to treat implicit disorders, such as Één klein probleempje and Zen Garden, we generally used a more abstract representation of player achievement, such as the growth of flowers in the garden or narrative progression in the story, as our design strategy in these gamifications had less emphasis on challenge.

**Structural game elements**

The structural game elements used in the design tended to be strongly influenced by the nature of disorder that the therapy seeks to address. Overall, the element of *progression* was commonly used as the structural game element in most of the gamifications which were cited as case studies. We felt that this element was appropriate as it helped emphasize the achievements and success of players in their therapeutic activity, replicating a commonly used strategy of highlighting past successes [60]. The *game leveling system* in ReadySetGoals and Addiction Beater was designed to enhance the experience of challenge which we felt appealed to people having substance abuse problems, who tended to favor sensation-seeking experiences, as such users also tended to be sensitive toward rewards (see [10]). The *player leveling system* was also used to increase the feeling of achievement from their successes in the activities. In Zen Garden, however, we felt that progression could add pressure for users to perform, especially for people with low self-esteem who were the main target audience of this gamification. Therefore, no performance-based progression element was used in this gamification.

To our knowledge this is the first paper in which the demands, restrictions, and possibilities of interdisciplinary health/game research are combined in a framework aimed to help designers integrate game design elements with therapeutic content in the mental health care context. Previous practice-based gamification approaches that have been proposed in the literature tend not to focus on a specific domain [63,64], or are designed for use in other areas such as business and education [65]. However, our method was developed specifically to support the design process of mental health care gamification, with the key processes used in a therapeutic activity forming the foundation of the concepts and models proposed in our framework. The Dual-Loop model serves as a practical tool to help designers deconstruct the processes used within a therapeutic activity and allow them to best determine the appropriate design strategy to create engaging experiences around each therapeutic component. The game therapy world concept provides a basic way for designers to analyze the potential impact of their gamification design on the therapeutic activity, an essential process which can be difficult to carry out in mental health gamification [21]. Moreover, our proposed method highlights how the game and therapeutic elements could be balanced in the development of a gamified intervention.

### Conclusion

In this paper, we propose a framework for the gamification of mental health care therapies. First, we analyzed existing mental health care gamifications and propose the concept of the game therapy world to illustrate how game elements could be integrated into therapeutic activities. We highlighted 4 different components of a game therapy world: the *performance space*, *rules*, *content*, and *structure*, which form a key part of a therapeutic activity that can be enhanced using game elements.

In addition, to aid developers in the process of designing the gamifications and allow them to better analyze the effect of their design choice on the 4 aforementioned components, we proposed the Dual-Loop Design model. This model consists of a core-game loop encompassing a therapeutic activity loop. The role of the designer is to consider how “presentation design,” “game logic design,” and “feedback design” could be used to enhance the “sample”, “user action,” and “therapy principle” components in the therapeutic activity loop. Further *structural game elements* such as player progression could then be added to encourage sustained engagement across the loops.

To illustrate how our framework could be used in practice, we provided 4 case studies of gamifications we developed and analyzed their design process through our proposed framework.

It should be noted, however, that there are several limitations to the framework proposed in this study. First, the framework itself was developed based on a limited class of intervention types and mental disorders. Whether the framework would be generalizable to other types of mental health intervention activities (eg, preventative health care or posttreatment activities) or psychological disorders remains unclear. In addition, the framework aims to support the design process of mental health gamifications formed from integrated therapy game worlds and not those formed from separated game and therapy worlds (such as in the “Integrating Game Elements Into Therapeutic Activity Through a Game Therapy Worlds Concept” section). Finally, the framework is conceptual in nature and specifying the tools and methods (prototype testing approaches, etc.) used in the design and development of the gamification elements (presentation, game logic, etc.) is beyond the scope of our framework.

In our future work, we would further investigate the general applicability of our models and framework toward gamifications in other domains, starting from the more general areas of health care (eg, gamification in preventative and posttreatment care). In addition, we would examine the tools and methods which could be used to support the design of gamifications based on our proposed Dual-Loop model and evaluate their effectiveness. In particular, we would investigate the various formative and summative evaluation approaches which could be effective in evaluating the gamification prototypes generated during different stages of design and conceptualization.

## Abbreviations

CBM: cognitive bias modification
CBM-I: cognitive bias modification of interpretations
COMET: Competitive Memory Training
